# Ferroptotic pores induce Ca^2+^ fluxes and ESCRT-III activation to modulate cell death kinetics

**DOI:** 10.1038/s41418-020-00691-x

**Published:** 2020-12-17

**Authors:** Lohans Pedrera, Rafael A. Espiritu, Uris Ros, Josephine Weber, Anja Schmitt, Jenny Stroh, Stephan Hailfinger, Silvia von Karstedt, Ana J. García-Sáez

**Affiliations:** 1 Institute for Genetics, University of Cologne, Joseph-Stelzmann-Straße 26, 50931 Cologne, Germany; 2 CECAD Cluster of Excellence, University of Cologne, Joseph-Stelzmann-Straße 26, 50931 Cologne, Germany; 3 Interfaculty Institute of Biochemistry, Eberhard-Karls-Universität Tübingen, 72076 Tübingen, Germany; 4 Department of Translational Genomics, Center of Integrated Oncology Cologne-Bonn, Medical Faculty, University of Cologne, Joseph-Stelzmann-Straße 26, 50931 Cologne, Germany; 5 Center for Molecular Medicine Cologne, Medical Faculty, University Hospital of Cologne, Robert-Koch-Straße 21, 50931 Cologne, Germany; 6 Present Address: Department of Chemistry, De La Salle University, 2401 Taft Avenue, Manila, 0922 Philippines

**Keywords:** Cell biology, Molecular biology

## Abstract

Ferroptosis is an iron-dependent form of regulated necrosis associated with lipid peroxidation. Despite its key role in the inflammatory outcome of ferroptosis, little is known about the molecular events leading to the disruption of the plasma membrane during this type of cell death. Here we show that a sustained increase in cytosolic Ca^2+^ is a hallmark of ferroptosis that precedes complete bursting of the cell. We report that plasma membrane damage leading to ferroptosis is associated with membrane nanopores of a few nanometers in radius and that ferroptosis, but not lipid peroxidation, can be delayed by osmoprotectants. Importantly, Ca^2+^ fluxes during ferroptosis induce the activation of the ESCRT-III-dependent membrane repair machinery, which counterbalances the kinetics of cell death and modulates the immunological signature of ferroptosis. Our findings with ferroptosis provide a unifying concept that sustained increase of cytosolic Ca^2+^ prior to plasma membrane rupture is a common feature of regulated types of necrosis and position ESCRT-III activation as a general protective mechanism in these lytic cell death pathways.

## Introduction

Ferroptosis is a caspase-independent form of regulated necrosis characterized by the generation of iron-dependent lipid peroxides in cellular membranes [1, 2]. Cells dying via ferroptosis are characterized by plasma membrane rupture and release of otherwise confined intracellular components including pro-inflammatory damage-associated molecular patterns [3]. Therefore, ferroptosis this type of cell death is associated with necroinflammation and with activation of the innate immune system. Ferroptosis has been shown to contribute to ischemia/reperfusion injury [4, 5], tissue damage, and organ demise [6], and to several other pathologies including neurodegenerative diseases and cancer [7]. For these reasons, understanding the molecular mechanisms governing membrane rupture during ferroptosis is not only of biological, but also of medical relevance.

In contrast to other cell death pathways that possess a specific agonistic cellular machinery to mediate cell death, ferroptosis seems to be executed upon inhibition of ferroptosis antagonists [8]. The best characterized trigger of ferroptosis is depletion or inhibition of glutathione peroxidase 4 (GPX4), a unique enzyme responsible for reducing peroxidized phospholipids in membranes to lipid alcohols [9, 10]. Treatment with Ras-selective Lethal small molecule 3 (RSL3), which directly inhibits GPX4, or with Erastin-1, which affects the activity of GPX4 by diminishing the levels of its co-substrate glutathione, is broadly used methods to induce ferroptosis [1, 11, 12].

A fundamental step in ferroptosis execution is the final disruption of the plasma membrane. However, the molecular mechanism causing the loss of plasma membrane integrity and the nature and size of the membrane injury in ferroptosis has remained unexplored. Damage to the plasma membrane in other forms of necrotic cell death such as necroptosis [13–15], pyroptosis [16, 17], or upon toxin-induced cell death [18] results in the activation of ion fluxes. In these scenarios, Ca^2+^ fluxes have been linked with the activation of membrane repair mechanisms [19–21]. The endosomal sorting complexes required for transport (ESCRT) machinery seems to play a critical counterbalancing role that can delay cell death in necroptosis and pyroptosis [17, 22, 23].

Here we investigated the molecular mechanism of plasma membrane permeabilization during ferroptosis triggered by Erastin-1 and RSL3 in various cellular models. Using live-cell imaging and flow cytometry, we tracked in parallel the kinetics of different hallmarks of ferroptosis. We find that lipid peroxidation during ferroptosis precedes a sustained increase of cytosolic Ca^2+^ and final plasma membrane breakdown. We also identify the formation of nanopores as a core mechanism that triggers plasma membrane burst during ferroptosis, which consequently can be inhibited by osmoprotectants of proper size. Finally, we link the increase in cytosolic Ca^2+^ in ferroptosis with the activation of the ESCRT-III machinery, which acts as a protective mechanism that delays cell death. This has immunological consequences, as depletion of ESCRT-III modulates the cytokine secretion from ferroptotic cells, thereby reshaping the inflammatory signature of ferroptosis. Our findings support a general role of the ESCRT-III machinery in counterbalancing membrane damage and in modulating the immunological outcome during regulated necrosis.

## Results

### Sustained increase in cytosolic Ca^2+^ is a hallmark of ferroptosis

**Fig. 1:**
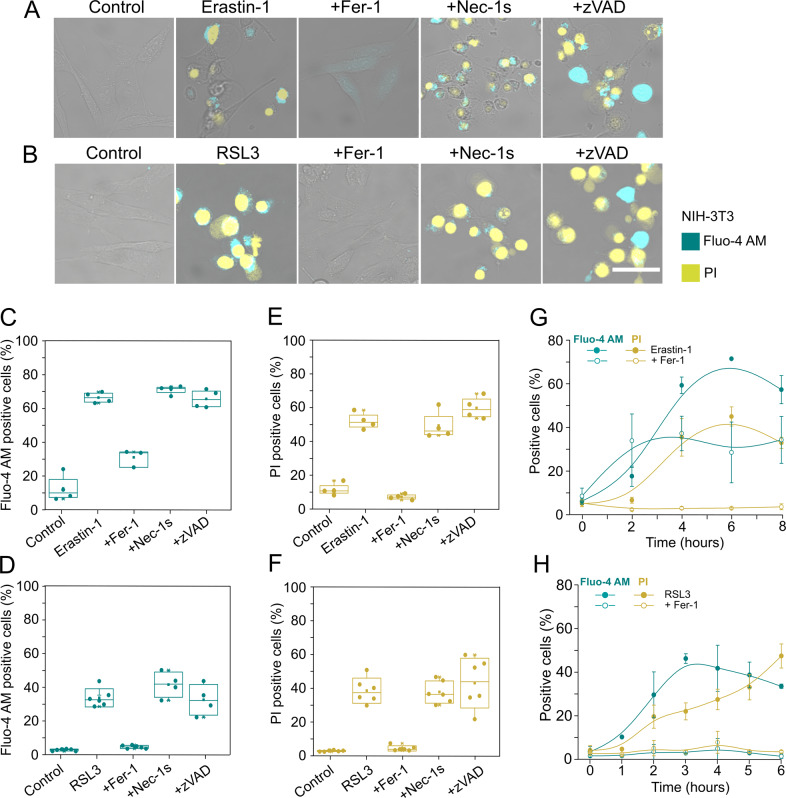
Cytosolic Ca^2+^ increases and cell death during ferroptosis in a treatment-dependent manner. Confocal images of NIH-3T3 cells treated with Erastin-1 (**A**) or RSL3 (**B**) in the presence or not of different cell death inhibitors. Pictures are representative of at least three independent experiments. Scale bar, 50 µm. Increase of Fluo-4 AM and PI-positive cells after 24 h of treatment with Erastin-1 (**C,** **E**) or RSL3 (**D,** **F**). Time course of the increase in the percentage of Fluo-4 AM and PI-positive cells upon treatment with Erastin-1 (**G**) or RSL3 (**H**), in the presence or not of Fer-1. The values represent the mean and the standard deviation of at least three independent flow cytometry experiments. An increase in cytosolic Ca^2+^ signal was detected using the Ca^2+^ indicator Fluo-4 AM, and plasma membrane breakdown was detected with PI. Fer-1 ferroptosis inhibitor ferrostatin-1, Nec-1s necroptosis inhibitor necrostatin-1s, zVAD pan-caspase inhibitor for apoptosis and pyroptosis. Concentrations: Erastin-1 (10 µM), RSL3 (2 µM), Fer-1 (2 µM), Nec-1s (10 µM), zVAD (20 µM).

Increase in cytosolic Ca^2+^, cell swelling, rounding, as well as plasma membrane breakdown have been described as hallmarks for necroptosis and pyroptosis [13, 24, 25]. In contrast to apoptosis, such increase in cytosolic Ca^2+^ is commonly associated with plasma membrane damage during these forms of regulated necrosis [26]. To assess whether these cellular alterations also take place during ferroptosis, we measured cytosolic Ca^2+^ levels in parallel to changes in cell morphology and plasma membrane breakdown by live-cell confocal imaging (Fig. 1A, B) and by flow cytometry (Fig. 1C–H) in mouse fibroblasts (NIH-3T3) treated with Erastin-1 or RSL3. We used fluo-4 acetoxymethyl (Fluo-4 AM) as a fluorescent form of the Ca^2+^ indicator [27], to visualize intracellular Ca^2+^ levels and the fluorescent DNA-intercalating agent propidium iodide (PI) as a marker of irreversible plasma membrane disruption and cell death [28].

We detected a clear increase in cytosolic Ca^2+^ concentration in NIH-3T3 cells upon treatment with Erastin-1 or RSL3 (Fig. 1A, B). This was accompanied by cell shape changes including cell rounding and the appearance of a single swelling bleb followed by a complete breakdown of plasma membrane integrity. Similar events were visualized when human fibrosarcoma cells (HT-1080) and human breast carcinoma cells (Mda-157) were treated with RSL3 (Fig. S1). In all cellular model systems, final membrane disruption resulted in the loss of intracellular Ca^2+^ and Fluo-4 AM dye, albeit with different kinetics and extension depending on the ferroptosis inducer used and cell line studied (Figs. 1 and S1). Increase in cytosolic Ca^2+^, cell rounding, and plasma membrane breakdown were specifically inhibited by the ferroptosis inhibitor ferrostatin-1 (Fer-1) [1], but not by the necroptosis inhibitor necrostatin-1s (Nec-1s) nor the pan-caspase inhibitor zVAD [13, 22], suggesting that all these events are hallmarks of ferroptosis (Fig. 1A–F). Similar to NIH-3T3 cells, Fer-1 also completely inhibited the increase in cytosolic Ca^2+^ and cell death in HT-1080 and Mda-157 cells upon treatment with RSL3 (Fig. S1).

To characterize the temporal relationship between cytosolic Ca^2+^ increase and plasma membrane disruption upon Erastin-1 or RSL3 treatment in the NIH-3T3 cells, we tracked the time course of Fluo-4 AM and PI fluorescence by flow cytometry in parallel (Fig. 1G, H). Independent of treatment, an increase in cytosolic Ca^2+^ took place hours before plasma membrane breakdown. In Erastin-1-treated cells, we found that cytosolic Ca^2+^ increase was not completely abolished by Fer-1 (Fig. 1A), with about 40% of the cell population remaining Fluo-4 AM positive (Fig. 1C, G). In contrast, Fer-1 completely prevented cytosolic Ca^2+^ increase when ferroptosis was induced with RSL3 (Fig. 1B, D, H). These results suggest that Erastin induces two distinct Ca^2+^ signaling processes: one that is unrelated to ferroptosis, and a later one, specific to ferroptosis. This late increase in cytosolic Ca^2+^ can be considered a hallmark of ferroptosis, as it was inhibited by Fer-1 and was common to Erastin-1 or RSL3-induced ferroptosis.

### **Lipid oxidation precedes the increase in cytosolic Ca**^***2+***^**and plasma membrane breakdown**

**Fig. 2:**
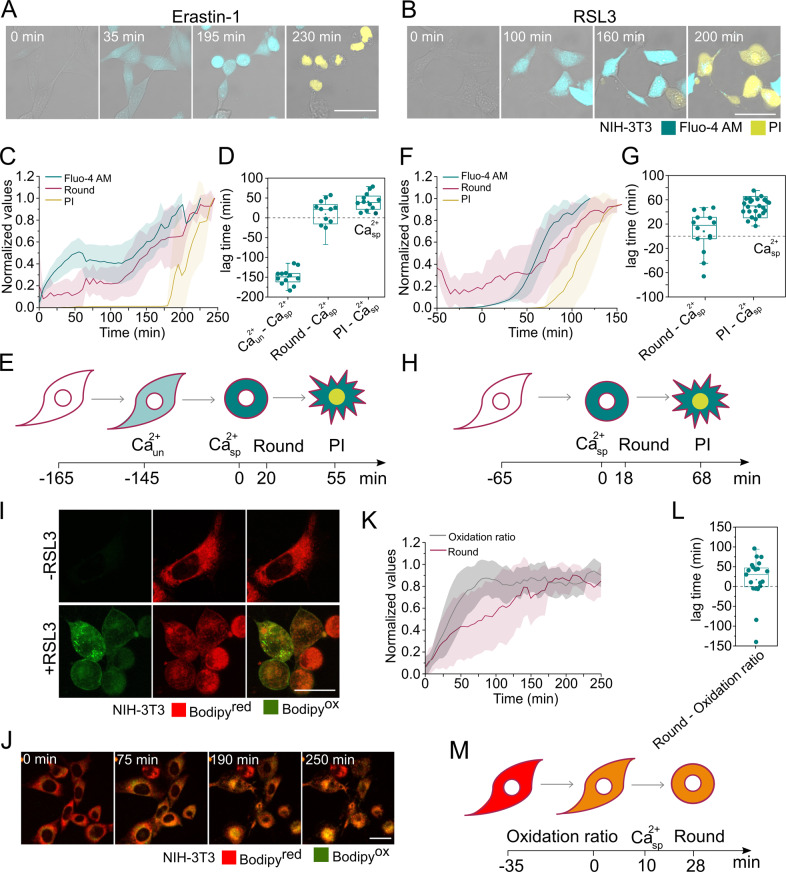
Increase in cytosolic Ca^2+^ and lipid oxidation take place prior to plasma membrane breakdown. Time-lapse images of NIH-3T3 cells treated with **A** Erastin-1 or **B** RSL3, and monitored for cell rounding and Fluo-4 AM and PI staining. Scale bar, 50 µm. **C** Kinetics of increase of the normalized Fluo-4 AM signal, cell rounding, and PI intake upon Erastin-1 treatment. Interval time between measurements was 5 min. Plots show the average (*N* = 16 cells) temporal relationships between the normalized parameters. All cells were synchronized to the first appearance of specific Ca^2+^ signal (*t* = 0). **D** Time delay between a specific Ca^2+^ flux and other ferroptotic events (unrelated Ca^2+^, change in cell rounding and PI) observed upon Erastin-1 treatment. **E** Graphical representation of the sequence of events observed during Erastin-1-induced ferroptosis. **F** Kinetics of increase of the normalized Fluo-4 AM signal, change cell rounding, and PI intake upon RSL3 treatment. Plots show the average (*N* = 25 cells) temporal relationships between the normalized parameters. All cells were synchronized to the first appearance of specific Ca^2+^ signal (*t* = 0). **G** Time delay between specific Ca^2+^ flux and other ferroptotic events (change in shape and PI) observed upon RSL3 treatment. Interval time between measurements was 5 min. **H** Graphical representation of the sequence of events observed during RSL3-induced ferroptosis. **I** Live cells confocal imaging of NIH-3T3 cells labeled with BODIPY before and after 2 h of treatment with RSL3. Pictures are representative of at least three independent confocal microscopy experiments. **J** Time-lapse images of lipid peroxidation in NIH-3T3 cells upon treatment with RSL3. Scale bar, 25 µm. **K** Time course of the increase of the normalized oxidation ratio and change in cell rounding upon RSL3 treatment. Plots show the average (n = 21 cells) temporal relationships between the normalized parameters. All cells were synchronized to the increase in the Oxidation ratio (*t* = 0). **L**) Time delay between cell round and oxidation ratio observed upon RSL3 treatment. **M** Graphical representation of the sequence of the lag time observed between oxidation ratio and other ferroptotic events during RSL3-induced ferroptosis. In **C**, **F**, and **K**, values were normalized between 0 and 1. In **D**, **G**, and **L**, *t*_50_ of each event was calculated from individual curves obtained per single cells. These values correspond to the time at 50% of the maximum signal. Concentrations: Erastin-1 (10 µM) and RSL3 (2 µM).

The detailed temporal relationship between ferroptosis hallmarks in bulk cell experiments is masked by the heterogeneity in which the cells in the population undergo ferroptosis upon treatment. To address this issue, we quantified the increase in Fluo-4 AM signal, cell rounding, and PI intake at the single-cell level using live-cell confocal microscopy. From the kinetic curves obtained from single cells (Fig. 2C, F, K), we calculated the time required to achieve 50% change of each ferroptotic phenotype (*t*_50_). This allowed us to set the lag time between each process in ferroptosis (Fig. 2D, G, L).

Independently of the ferroptotic trigger used, an increase in cytosolic Ca^2+^ preceded cell rounding and complete plasma membrane collapse (Fig. 2A, B). However, as suggested by the flow cytometry experiments (Fig. 1G), the increase in cytosolic Ca^2+^ in the presence of Erastin-1 was a two-step process with biphasic behavior (Fig. 2C). The first event reached saturation around 40% of the maximum Ca^2+^ signal and corresponded to the unrelated calcium (Ca^2+^_un_) increase, as it was not inhibited by Fer-1 (Fig. 1G). The second Ca^2+^ rise, common to RSL3 treatment and inhibited by Fer-1 (Figs. 1H and 2F) corresponded to the cytosolic Ca^2+^ increase specific to ferroptosis (Ca^2+^_sp_). Temporally, Ca^2+^_un_ increased happened after Erastin-1 treatment, followed by Ca^2+^_sp_ rise, cell rounding, and final PI intake (Fig. 2C–E). In contrast, RSL3-treated cells were characterized by a unique and specific Ca^2+^ rise event followed by cell rounding and final PI intake (Fig. 2F–H). Given the fact that RSL3 induced only the ferroptosis-specific Ca^2+^ signal, we selected this treatment to characterize the connection of this ion with lipid peroxidation and membrane damage during ferroptosis.

To visualize lipid peroxidation in membranes we used the lipid peroxidation sensor C11 BODIPY 581/591. RSL3 treatment indeed promoted lipid peroxidation at the plasma membrane as well as in subcellular membranes, as detected by the increase in the green fluorescent signal of BODIPY (BODIPY^ox^) (Fig. 2I). We next tracked in parallel the change in the oxidation ratio and cell rounding (Fig. 2J, K). Lipid peroxidation preceded the rounding of the cells. Considering that the lag time between the increase of cytosolic Ca^2+^ and cell rounding is consistently lower than the lag time between lipid peroxidation and cell rounding, we could estimate that the rise in Ca^2+^ takes place after lipid peroxidation (Fig. 2M). This estimation was based on extrapolation between the independent kinetics of the increase of the Fluo-4 AM (Fig. 2F–H) and BODIPY oxidation ratio (Fig. 2K, L).

### Osmotically active agents protect cells against ferroptosis

**Fig. 3:**
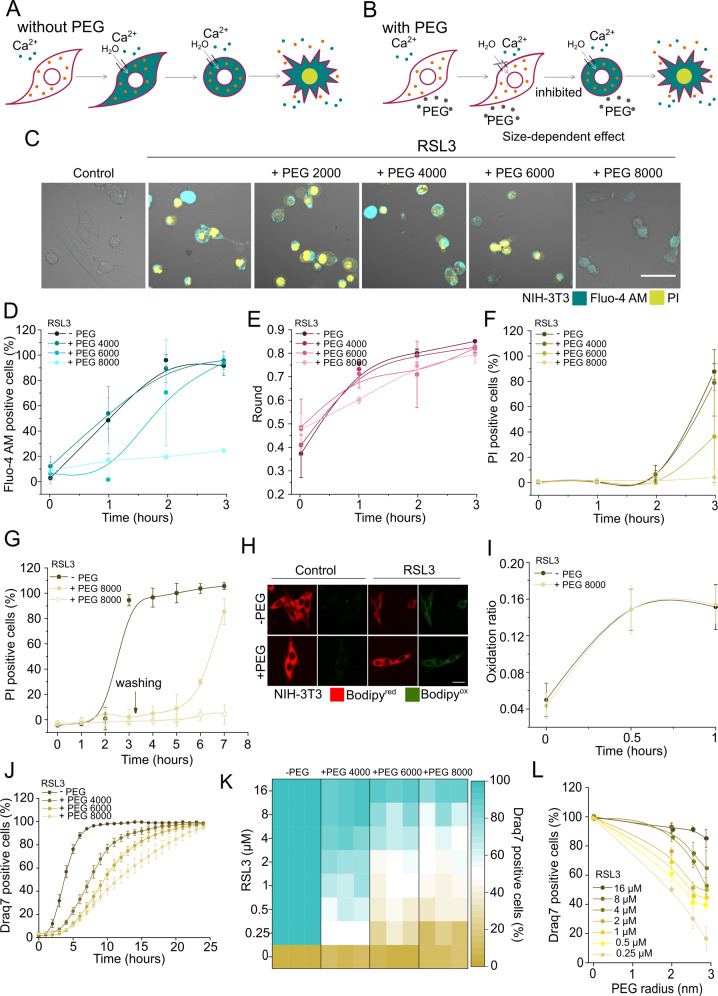
PEGs of high sizes provide osmotic protection against ferroptosis. **A** Impermeant intracellular molecules impose an osmotic gradient after pore opening that leads to net influx of water molecules and cell lysis. **B** PEGs of proper size can prevent this effect if their size is large enough to do not cross the membrane through the pores. **C** Live cells confocal images of the effect of PEGs of different sizes on RSL3-induced (2 µM) ferroptosis at 3 h of treatment. Pictures are representative of at least three independent experiments. Scale bar, 50 µm. **D** Time course of increase of Fluo-4 AM fluorescent signal **E** change in cell rounding, and **F** PI intake in NIH-3T3 cells treated with RSL3 (2 µM), in the presence or not of PEGs of different sizes. Each data point represents the mean of at least six replicas made in three independent confocal microscopy experiments. At least 100 cells were analyzed for each replica. **G** Recovery of cell death after washing out PEG 8000. NIH-3T3 cells were treated with RSL3 (2 µM) in the presence of PEG 8000. After 3 h, the media containing osmoprotectant and RSL3 was exchanged by fresh media, and the kinetic of cell death was tracked over time. **H** Representative images of the effect of PEG 8000 in the oxidation ratio of C11 BODIPY 581/591 on RSL3-induced (2 µM) ferroptosis at 1 h of treatment in NIH-3T3 cells. Scale bar, 25 µm. **I** Oxidation ratio of C11 BODIPY 581/591 in cell treated with RSL3 (2 µM) in the presence or not of PEG 8000. Each data point represents the mean of at least five replicas made in three independent confocal microscopy experiments. At least 100 cells were analyzed for each condition. **J** Long-time kinetics of cell death of NIH-3T3 cells treated with RSL3 (4 µM), in the presence or not of PEGs of different sizes. **K** Cell death heat map of the effect of different PEGs on the extension of cell death (12 h) in NIH-3T3 treated with different concentrations of RSL3. **L** Percentage of dead cells as a function of PEG size upon 12-h treatment of NIH-3T3 cells with different RSL3 concentrations.

Pore formation is a common feature in different types of regulated cell death including apoptosis [29], necroptosis [13], and pyroptosis [30]. The opening of plasma membrane pores leads to a net influx of water molecules as a consequence of the osmotic imbalance resulting from the high concentration of large intracellular molecules that cannot pass through membrane pores (Fig. 3A). Such an effect can be prevented by the addition of osmoprotectants of appropriate size that are not able to enter the cell through the pores, thus counterbalancing the intracellular osmotic pressure, water influx, and the consequent cell collapse (Fig. 3B) [31, 32]. According to this, the permeability of membrane pores, but not of ion channels, can be modulated by the osmotic effects exerted by polyethyleneglycols (PEGs) [13, 30, 31]. To investigate whether the plasma membrane permeabilization observed in ferroptotic cells was connected with membrane pores, we assessed the effect of PEGs of different sizes on the kinetics and extent of different ferroptotic hallmarks.

We used the following PEGs (in parenthesis their hydrated radii as reported in refs. [31, 33]): 400 (0.56 nm), 600 (0.69 nm), 1000 (0.94 nm), 2000 (1.6 nm), 4000 (1.8 nm), 6000 (2.3 nm), and 8000 (2.7 nm). The addition of smaller PEGs up to 4000 did not prevent the increase of cytosolic Ca^2+^, cell rounding or cell death (Figs. 3C–F and S2). In contrast, both the increase in cytosolic Ca^2+^ levels and cell death were delayed in the presence of higher-molecular weight PEGs (6000 and 8000) in a size-dependent manner (Figs. 3D, F and S2D). In the presence of PEG 8000, we also observed a delay in the kinetics of cell rounding (Fig. 3E). These results indicate that the increase in cytosolic Ca^2+^, cell rounding, and plasma membrane breakdown are all events driven by osmotic forces. Notably, the protective effect of PEG 8000 in ferroptosis was reverted upon washing, which indicates that the membrane damage was stable over time (Fig. 3G). We also found that PEG 8000 did not prevent lipid peroxidation in RSL3-treated NIH-3T3 cells (Fig. 3H, I). These results confirmed that the effect of PEG 8000 was not a consequence of its ability to block the ferroptotic pathway, but rather the result of counterbalancing the water and ion fluxes resulting from pore formation in the plasma membrane.

Of note, for all the PEG sizes used, cell death was recovered when NIH-3T3 cells were treated for a longer period of time (24 h) (Fig. 3J). The inhibitory effect of PEGs on the kinetics of ferroptosis also decreased with RSL3 concentration (Figs. 3K, L and S3A). A similar effect was observed when different cell lines were treated with different concentrations of RSL3 in the presence or not of PEGs 4000, 6000, and 8000 (Fig. S3). Altogether, these results suggest that the extent of membrane damage (size and/or number of membrane pores) in ferroptosis increases with the time and the intensity of the stimulus. Using as a reference the PEG size that was able to protect at the lower concentrations of RSL3, we could roughly estimate the smaller size of the perforated sections of the plasma membrane to be around 2.5-nm radius (Fig. 3L). Altogether, our data support a general model of plasma membrane disruption in ferroptosis through the formation of pores of a few nanometers in radius.

### **ESCRT-III complex is activated during ferroptosis and antagonizes cell death**

**Fig. 4:**
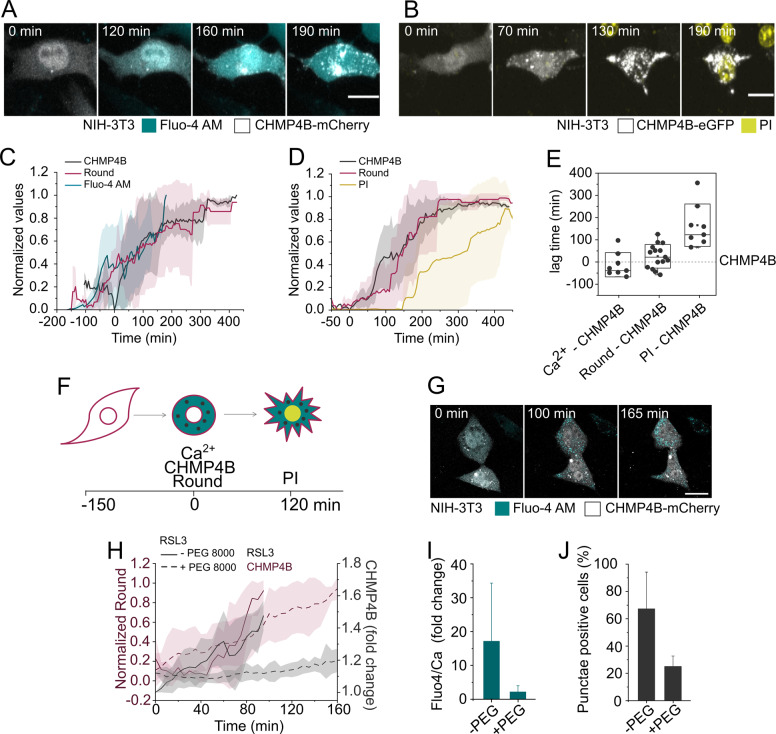
Membrane damage correlates with CHMP4B activation. Time-lapse live confocal images of NIH-3T3 cells transiently transfected with **A** CHMP4B-mCherry or **B** CHMP4B-eGFP, treated with RSL3, and monitored for CHMP4B puncta formation, Fluo-4 AM, and PI staining. Scale bar, 20 µm. **C**, **D** Kinetics of the appearance of CHMP4B puncta and increase of cytosolic Fluo-4 AM signal, change in cell rounding, and PI intake upon RSL3 treatment. Plots show the average temporal relationships between normalized **C** mCherry (*N* = 8 cells) or **D** eGFP (*N* = 15 cells) fluorescence intensity standard deviation and cell rounding. All cells were synchronized to the first appearance of CHMP4B puncta (*t* = 0). Interval time between measurements was 5 min. **E** Time delay between the appearance of CHMP4B puncta, Ca^2+^ signal, cell rounding, and PI. *t*_50_ of each event was calculated from individual curves obtained per single cells (shown in **C** and **D**). These values correspond to the time at 50% of the maximum signal and were plotted as lag time with respect to appearance of the CHMP4B puncta. **F** Graphical representation of the sequence of events related to CHMP4B activation in ferroptosis. **G** Time-lapse images of NIH-3T3 cells transiently transfected with CHMP4B-mCherry and treated with RSL3 (2 µM) in the presence of PEG 8000. Scale bar, 15 µm. **H** Kinetics of the appearance of CHMP4B puncta and change in cell rounding upon RSL3 treatment in the presence or absence of PEG 8000. Plots show the average temporal relationships between normalized cell rounding and the folding increase of the standard deviation of mCherry (*n* = 8 cells in the experiment with PEG 8000 and 6 cells in the experiment without PEG 8000). The activation of ESCRT-III was measuring as the fold increase of the SD of the mCherry CHMP4B fluorescence signal over time. Interval time between measurements was 5 min. **I** Fold increase of the Fluo-4 AM mean intensity and percentage of cells containing CHMPH4 puncta in the presence or absence of PEG 8000.

It has been previously reported that during necroptosis and pyroptosis, membrane repair dependent on the action of the ESCRT-III complex delays cell death [17, 22, 23]. To investigate the role of ESCRT-III complex in ferroptosis, we transiently transfected NIH-3T3 cells with the component CHMP4B, tagged with either eGFP or mCherry, and monitored its dynamics using live-cell confocal imaging. It is well accepted that ESCRT-III can mediate plasma membrane repair by different mechanisms that include membrane shedding and endocytosis [18, 34]. In the first case, ESCRT-III is recruited to the plasma membrane but in the second case, damaged membrane sections are removed by endocytosis and the ESCRT machinery is recruited at later stages to the endosomes [18, 34]. Even though in our experiments we cannot distinguish between these two scenarios, we used CHMP4B puncta formation as a proxy for ESCRT-III activation. Initially, CHMP4B mostly showed homogenous cytosolic distribution, however, upon RSL3 treatment, the protein localized to distinct puncta that increased in number and fluorescence intensity over time (Figs. 4A, B and S4). This observation was similar to the reported behavior of CHMP4B during necroptosis [22, 23] and pyroptosis [17], and indicates that the ESCRT-III complex is activated during ferroptosis.

We next quantified the temporal correlation of CHMP4B puncta formation with the increase in cytosolic Ca^2+^ concentration, cell rounding, and final plasma membrane breakdown at the single-cell level by live-cell confocal imaging. CHMP4B puncta formation coincided approximately with the increase in cytosolic Ca^2+^ concentration (Figs. 4C–E and S4). This is consistent with the fact that repair mechanisms are activated shortly after the rise in cytosolic Ca^2+^ concentration [19, 20], and also with previous results showing the formation of CHMP4B puncta after membrane damage [17, 22, 23, 34]. Furthermore, these results showed that cells round up at about the same time of these two events (Fig. 4C–E), consistent with a putative role of osmotic forces. Final membrane breakdown occurred after a sustained increase in cytosolic Ca^2+^ levels, cell rounding, and ESCRT-III complex activation, likely when the cellular repair machinery was ultimately overwhelmed (Fig. 4B, E, F).

To get further insight into the link between membrane damage and the role of Ca^2+^ in the activation of ESCRT-III during ferroptosis, we followed the increase in cytosolic Ca^2+^ in parallel with cell rounding and CHMP4B distribution in NIH-3T3 cells co-treated with RSL3 and PEG 8000, using live-cell confocal microscopy. This experiment was based on our findings that treatment with PEG 8000 blocks Ca^2+^ fluxes and ferroptosis progression, but does not affect lipid peroxidation nor cell rounding. Notably, treatment with PEG 8000 also blocked CHMP4B puncta formation, probably as a consequence of inhibiting Ca^2+^ fluxes (Fig. 4G–J). These results strengthen a model in which ESCRT-III complexes are activated in a Ca^2+^-dependent manner to repair membrane damage.

Interestingly, the early increase in cytosolic Ca^2+^ observed in Erastin-treated NIH-3T3 cells took place before cell rounding and CHMP4B puncta formation (Fig. S5), suggesting that the early increase in cytosolic Ca^2+^ during Erastin treatment is unrelated to plasma membrane damage.

**Fig. 5:**
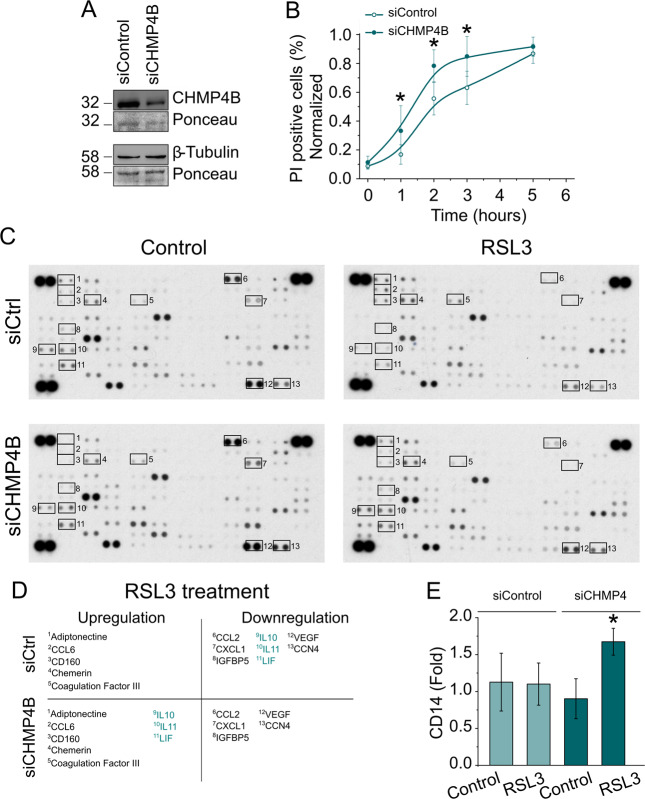
ESCRT-III modulates cell death and the immunologic outcome of ferroptotic cells. **A** Immunoblot showing the knock down of CHMP4B in NIH-3T3 cells. **B** Effect of knocking down CHMP4B on the kinetics of ferroptosis. **C** Effect of CHMP4B depletion on the cytokine profile of ferroptotic cells. NIH-3T3 cells were treated with RSL3 (2 µM) for 24 h. Supernatants were filtered, and the cytokine composition was analyzed by the proteome profiler mouse XL cytokine Array kit. **D** Summary of the cytokines that are regulated in **C**. Cytokines that are differently regulated upon CHMP4B depletion are highlighted in green. **E** CD14 expression in the plasma membrane of raw 264.7 macrophages after stimulation with the supernatants obtained from NIH-3T3 cells treated with RSL3, as indicated in **C**. Each bar represents the mean and standard deviation for three independent experiments. The statistical comparison was carried out by the Student’s *t*-test. * indicates independent groups with significant differences among them (*p* < 0.05).

To examine the functional role of ESCRT-III in ferroptosis, we assessed the effect of knocking down CHMP4B on cell death in NIH-3T3 cells (Fig. 5A, B). We observed a higher and faster incidence of cell death in CHMP4B knockdown cells compared with the control, particularly at early time points (1–3 h), indicating that the loss of CHMP4B results in higher sensitization of the cells toward ferroptosis. Importantly, these findings show that the ESCRT-III machinery plays a role in protecting cells against ferroptosis most likely by counterbalancing membrane damage.

During necroptosis and pyroptosis, the ESCRT-III machinery has been shown to regulate the release of cytokines and other inflammatory factors, which affects the communication with immune cells [17, 22, 23, 35]. Thus, we set out to determine whether ferroptosis can directly modulate cytokine secretion in different cell lines and upon different treatments using the proteome profiler XL cytokine Array kit (Figs. 5C and S6). Upon ferroptosis induction, we detected changes in the levels of a subset of cytokines that appeared cell line- and treatment-dependent. Furthermore, knocking down CHMP4B altered the cytokine profile obtained after treatment with RSL3 (Fig. 5C, D) or Erastin-1 (Fig. S6C). These results suggest that ESCRT-III generally impacts cytokine secretion in ferroptotic cells, albeit the concrete cytokines altered are treatment-dependent.

Next, we explored the role of ESCRT-III-modulated membrane repair in the regulation of a potential immune response. To this end, we collected supernatants from NIH-3T3 cells with control or CHMP4B knockdown and treated with DMSO or RSL3. We spiked supernatants after collection with Fer-1 to prevent that they induced cell death in later experiments due to residual RSL3 in the media. These supernatants were then used to treat immortalized bone-marrow-derived macrophages. To monitor macrophages activation, expression of the CD14 was analyzed by flow cytometry. Strikingly, only supernatants from RSL3-treated CHMP4B-silenced cells were capable of increasing CD14 surface expression of mouse macrophages (Fig. 5E). Altogether these results indicate that similar to necroptosis and pyroptosis, ESCRT-III also modulates the immunological output of ferroptotic cells.

## Discussion

Despite the rapid progress made in recent years on the understanding of ferroptosis, how the final plasma membrane rupture is executed remains unclear. Here we examined the cellular alterations during ferroptosis execution, including an increase in cytosolic Ca^2+^, cell rounding, and lipid peroxidation, and correlated them with plasma membrane disruption. We found that GPX4 inactivation induces an increase in cytosolic Ca^2+^ that precedes complete plasma membrane disruption. Unlike lipid peroxidation, both events are driven by osmotic forces and linked to the formation of pores of few nanometers in the plasma membrane. A sustained increase in cytosolic Ca^2+^ is necessary for the activation of the ESCRT-III machinery for membrane repair, which delays the kinetics of cell death likely by counterbalancing membrane damage upon ferroptosis. Importantly, activation of ESCRT-III during ferroptosis modulates the inflammatory microenvironment of the dying cells, which is a common feature with necroptosis [22, 23, 35] and pyroptosis [17].

Alteration of plasma membrane permeability is a feature of ferroptosis that is common with necroptosis and pyroptosis [13, 18, 24, 30, 36]. In necroptosis and pyroptosis, membrane permeabilization is mediated by dedicated protein cell-death effectors encoded in the genome [30, 36, 37]. In contrast, the protein- or lipid-based nature of the pores during ferroptosis execution is a key open question in the field. In this regard, it is a matter of strong debate whether peroxidation of PUFAs-PL at the plasma membrane would be sufficient to cause its permeabilization. It has been hypothesized that peroxidized phospholipids and their derivate lysophospholipids could be directly toxic to cells through the formation of small discontinuities in the plasma membrane involving membrane thinning and increase of membrane curvature [38]. Alternatively, lipid peroxidation could modulate the activity of membrane proteins, indirectly triggering membrane disruption. Here we found that lipid peroxidation occurred as an early event during ferroptosis progression, prior to markers of membrane damage like increased cytosolic calcium and cell rounding. We not only detected lipid peroxidation at the plasma membrane, but also in other intracellular membranes. This opens the question about the contribution of the subcellular localization of lipid peroxidation to ferroptosis progression.

We also identified that final plasma membrane disruption in ferroptosis is caused by an imbalance in osmotic forces due to the opening of small nanopores. Activation of osmotic forces leading to anion imbalance and cell bursting represents a common feature with necroptosis and pyroptosis [13, 30]. In this scenario, our results position the opening of pores at the plasma membrane as a core mechanism responsible for the execution of different types of regulated necrosis.

Different ion fluxes take place upon membrane damage, of which Ca^2+^ has a critical role linking the increase in plasma membrane permeability to the activation of intracellular signaling pathways [19, 39–41]. In this context, a rise in cytosolic Ca^2+^ has been related to the execution of different cell death processes including apoptosis, necroptosis, and pyroptosis, or unregulated necrosis [18, 39, 41, 42]. Previous work on the process of oxytosis, a type of cell death related to ferroptosis, connected lipid peroxidation with the increase of Ca^2+^ fluxes through specific channels [43]. Contradictory observations also suggested that Ca^2+^ has no impact on ferroptosis [1, 44] or linked to extracellular Ca^2+^ influx and oxidative cell death with the inhibition of the *X*_c_ system by glutamate [45]. Here we show that when ferroptosis is triggered by Erastin-1, there is an early increase in cytosolic Ca^2+^ event that takes place quickly after treatment and prior to ESCRT-III activation, but is not inhibited by Fer-1. This is followed by a second increase in cytosolic Ca^2+^ that is common with the single Ca^2+^ influx event observed in RSL3-induced ferroptosis and that can be inhibited by Fer-1. Our findings could therefore reconcile possible contradictions about the essential role of Ca^2+^ in ferroptosis, since the type of fluxes that is activated downstream GPX4 inactivation strongly depends on the treatment that is used. The early Ca^2+^ increase activated by Erastin-1 signaling could be triggered by the inhibition by Erastin-1 of the voltage-dependent anion-selective channel proteins 2 and 3 [1, 46], which are involved in the regulation of cellular Ca^2+^ homeostasis [47]. In contrast, ferroptosis-specific increase in Ca^2+^ observed upon Erastin-1 and RSL3 treatments are sustained and linked to membrane damage, as it was inhibited by osmotically active agents. We cannot discard the possibility that it is also a result of the indirect activation of endogenous Ca^2+^ channels, a process that is also connected to membrane damage [15, 48, 49]. Further work will be necessary to identify the cellular components that mediate ferroptosis-related Ca^2+^ fluxes and their role in ferroptosis progression.

Recently, the ESCRT-III complex has been identified as common machinery that mediates plasma membrane repair during necroptosis and pyroptosis [17, 22, 23]. ESCRT-III mediates the shedding and trapping of the damaged sections of the membranes into intracellular or extracellular vesicles in response to the increase of cytosolic Ca^2+^ [17, 22, 23]. As a result, the ESCRT-III machinery regulates cell death and impacts on the signals released from dying cells and on the activation of the immune system [17, 22, 23, 35]. Here we also found that the ESCRT-III machinery is relevant to protect cells against ferroptosis. We causally linked the activation of ESCRT-III with the increase in cytosolic Ca^2+^ upon membrane damage in ferroptosis and showed that ESCRT-III depletion via CHMP4B downregulation accelerates the kinetics of ferroptosis. This is in agreement with a recent study showing that knock down of CHMP5 or CHMP6 sensitizes cancer cells to ferroptosis [50].

The lag time between membrane damage and cell bursting represents a time frame in which the cells can communicate with the microenvironment [18]. By modulating the kinetics of cell death, the ESCRT-III machinery might thus affect the inflammatory factors that are released from ferroptotic cells, as previously proposed for necroptosis and pyroptosis [17, 22, 23, 35]. In agreement with this, we discovered that ESCRT-III affects the cytokine profile secreted from ferroptotic cells as well as the activation of macrophages exposed to ferroptotic supernatants. Our findings expand the role of ESCRT-III as a general machinery for counterbalancing regulated necrosis and its inflammatory consequences.

In summary, here we defined the cellular hallmarks of ferroptosis associated with plasma membrane permeabilization upon GPX4 inactivation. Activation of the ferroptotic pathway leads to lipid peroxidation in cellular membranes and a sustained increase of cytosolic Ca^2+^ levels as a result of membrane damage that precedes final cell bursting. These processes are governed by osmotic pressure and result from the formation of small pores of a few nanometers in the plasma membrane. The activation of Ca^2+^ fluxes due to membrane damage induces the activation of the ESCRT-III machinery, which counterbalances ferroptosis to modulate the kinetics of cell death. Our results that ESCRT-III modulates the cytokine levels in the ferroptotic microenvironment reveal the role of this machinery in the modulation of the inflammatory outcome of ferroptosis. These findings support a general model in which plasma membrane pore formation and membrane repair emerge as central and opposite mechanisms that control the progression of different types of regulated necrosis and their inflammatory signature.

## Materials and methods

### Reagents and antibodies

Erastin-1, RSL3, Fer-1, and Nec-1s were purchased from Biomol (Germany). PI and all PEGs were from Sigma-Aldrich (Germany). zVAD was obtained from APEXBIO (Houston, TX, USA). Fluo-4-AM and C11 BODIPY 581/591 were provided by Thermofisher (Germany). CHMP4B siRNA (L-018075-01-005) was purchased from Dharmacon (Germany). The CHMP4B-eGFP and CHMP4B-mCherry constructs were kindly provided by Prof. Christian Wunder (Institut Curie, Paris, France). Anti-CD14 (lone Sa14-2, 123307-123308) and isotype control antibodies were purchased from Biolegend. Anti CHMP4B (ab7291) was purchased from Abcam, and anti β-tubulin (sc-55529) was from Santa Cruz Biotechnology.

### **Cell lines, culture conditions, and transfection**

NIH-3T3 cells were obtained from Prof. Dr Andreas Linkermann (University Hospital Carl Gustav Carus at the Technische Universität Dresden). HT-1080 and Mda-157 cells were obtained from Dr Marcus Conrad (Helmholtz Zentrum München) under the terms of a material transfer agreement. Immortalized mouse bone-marrow-derived macrophages were kindly provided by Prof. Eicke Latz (Institute of Innate immunity, Bonn, Germany). Mouse embryonic fibroblasts (MEFs) were kindly provided by Prof. Manolis Pasparakis (University of Cologne, Germany), H441 cells were kindly provided by Prof. Julian Downward (The Francis Crick Institute, London, UK). All cell lines were tested for mycoplasma contamination. All cells were cultured in low glucose DMEM (Sigma, Germany) supplemented with 10% FBS and 1% penicillin–streptomycin (ThermoFisher, Germany), and grown in a humidified incubator containing 5% CO_2_ at 37 °C. The cells were frequently passaged at sub-confluence, and seeded at a density of 0.5–5 × 104 cells/mL. For knockdown experiments, cells were transfected with Lipofectamine 2000 (Invitrogen, Germany) with specific siRNA, for 48 h in 6-well (western blot) or 12-well (FACS) plates.

### **Flow cytometry measurements**

The plasma membrane integrity was tested by flow cytometry measuring the ability of cells to exclude PI. Flow-cytometric analyses were conducted using CytoFlex and data analyzed using the FACSDiva software (Beckman Counter, Germany). After treatment, both attached and nonattached cell populations were collected. Cells were washed twice with cold PBS, centrifuged (2500 *g*, 5 min, 4 °C), and resuspended in PBS containing Fluo-4-AM (1 µM). After 30 min of incubation at 37 °C, cells were washed, resuspended in PBS containing PI (2 µg/mL), and a total of 10,000 cells were analyzed by flow cytometry. PI fluorescence was determined in the PE-A channel and Fluo-4 AM/Ca in the FITC-channel. For PI, the distribution of fluorescence intensity in the negative control sample with living cells and in the positive control sample with dead cells did not overlap and we chose a threshold value intermediate between the well-resolved peaks that yielded 10% or less dead cells in the negative control. For Fluo-4 AM, the distribution of fluorescence intensity in the negative (living cells) and positive (dead cells) control populations partially overlapped and we chose a threshold value based on the negative control sample (living cells) using the geometric triangle method [51]. The triangle method constructs a line between the histogram peak and the farthest end of the histogram. This typically yielded values between 5 and 10% Fluo-4 AM/Ca positive cells in the control population.

### **CD14 stainings in immortalized BMDMs**

Supernatants from control or siCHMP4 cells treated with DMSO or RSL3 were collected and subsequently spiked with Ferrostatin-1 to prevent RSL3-induced cell death in subsequent co-cultures. 500,000 immortalized BMDMs (CL13) were seeded in six-well plates and stimulated for 24 h with these supernatants. Cells were then detached by scraping, washed with PBS, and incubated with FITC-coupled anti-CD14 antibody (clone Sa14-2, Bioloegend) or FITC-coupled isotype control at 1:100 in 100 µl for 1 h at room temperature. Next, samples were washed twice with in PBS, 2% FCS and FITC mean fluorescence intensity was determined by flow cytometry using a FACS Fortessa (BD) and the corresponding Diva (8.0) software. Subsequent data analysis was performed using FlowJo version 10.4.2.

### Bright-field and confocal microscopy

Cells were seeded in DMEM in IBIDI eight-well chambers (Ibidi, Germany) 24 h before the experiment. The day after, cells were washed with PBS to replace the media by phenol red-free DMEM (Sigma-Aldrich, Germany) supplemented with FBS and antibiotics. Cells were loaded with 1-µM Fluo-4 AM, 2-µg/mL PI, or 1 µM of C11 BODIPY 581/591 for 30 min at 37 °C. All images were acquired with a Zeiss LSM 710 ConfoCor3 microscope (Carl Zeiss, Jena, Germany) or a gSTED Leica confocal microscopy equipped with incubator at 37 °C and 5% CO_2_. Time-lapse imaging with z-stack acquisition was carried out before and after ferroptosis induction. Transmitted light and fluorescence images were acquired through a Zeiss C-Apochromat 40X, NA = 1.2 water immersion objective or a 63X, NA = 1.2 oil immersion objective onto the sample. Excitation light came from Argon ion (488 nm) or HeNe (561 nm) lasers. All images were processed in Fiji. The percentage of Fluo-4 AM-positive cells was calculated at each time point. For this, individual cells were automatically detected based on the fluorescence of the Fluo-4 AM. To define cells as positive, we arbitrarily set a fluorescence threshold in which the percentage of Fluo-4 AM-positive cells did not exceed 5% in the negative control. The total number of cells in each condition was determined in the transmitted light images.

### IncuCyte measurements

Kinetics of cell death were collected using the IncuCyte bioimaging platform (Essen). For this, cells were seeded in 96-well plates (10^4^ cells per well) 1 day before treatment. After treatment, four images per well were captured, analyzed, and averaged. Cell death was measured by the incorporation of DRAQ7. Data were collected as count of Draq 7 positive cells per total number of cells in each conditions and normalized taking as 100% the total count of cells in each condition.

### Lipid peroxidation measurements

The oxidation ratio of C11 BODIPY 581/591 was calculated as an indicator of lipid peroxidation per cell following the equation:

$${\mathrm{Oxidation}}\;{\mathrm{ratio}} = {\mathrm{Bodipy}}^{{\mathrm{ox}}}/({\mathrm{Bodipy}}^{{\mathrm{ox}}} + {\mathrm{Bodipy}}^{{\mathrm{red}}}),$$

where Bodipy^red^ corresponds to the non-oxidized fraction of the probe and was estimated based on the fluorescence intensity per pixel from the red image and Bodipy^ox^ corresponds to the oxidized fraction and was calculated based on the fluorescence intensity per pixel from the green channel fluorescence images. Those values were estimated based on the fluorescence intensity per pixel from the red and green channels in the fluorescence images (using Fiji). *t*_50_ values of the oxidation ratio per cell were calculated by adjusting the increase in the oxidation ratio over time with an exponential function (using Origin 8.0).

### Effect of osmotically active agents

The effect of PEGs of different sizes was assessed using confocal microscopy or Incucyte measurements. PEGs were solubilized in DMEM media and filtered before use. For the experiments, ferroptosis was induced in the presence of PEG (10 mM). We controlled that at this concentration the different PEGs were not toxic to the cells. For the washing experiments, the media containing PEG 8000 and RSL3 were exchanged by fresh media without them, after 3 h of treatment. At this time, we controlled that cells were still alive due to osmotic protection. From this point, we recorded the recovery of the kinetics of cell death after washing out.

### Cytokine arrays

Culture supernatants from cells treated with RSL3 or Erastin-1 for 24 h were analyzed for secreted cytokines/chemokines using Proteome Profiler mouse and human XL Cytokine Array (R&D Systems) according to the manufacturer’s instructions.

### Statistical methods

All measurements were performed at least three times and results are presented as mean ± standard deviation.

## Supplementary information

Suplementary figure legend

Figure S1

Figure S2

Figure S3

Figure S4

Figure S5

Figure S6
