# Supplementary material for: Ferroptotic pores induce Ca2+ fluxes and ESCRT-III activation to modulate cell death kinetics
Source: Cell Death Differ. 2020 Dec 17;28(5):1644–57. doi: 10.1038/s41418-020-00691-x (PMC8167089; doi:10.1038/s41418-020-00691-x)
Supplement: Supplementary file 1 — Suplementary figure legend [file 41418_2020_691_MOESM1_ESM.docx]

## Supplementary figure legends

**Figure S1: Cytosolic Ca^2+^ increase and cell death during ferroptosis in RSL3-treated cells.** A and B) Confocal images of cells treated with RSL3 in the presence or not of Fer-1. Pictures are representative of at least three independent experiments. Scale bar, 50 µm. C and D) Time course of the increase of cytosolic Fluo-4 AM and PI positive cells upon treatment with RSL3 in the presence or not of Fer-1. The values represent the mean and the standard deviation of at least three independent confocal microscopy experiments.

**Figure S2: PEGs of small sizes did not provide osmotic protection against ferroptosis.** A) Time course of the increase of cytosolic Ca^2+^, B) change in cell Round, and C) PI intake in NIH-3T3 cells treated with RSL3, in the presence or not of PEGs of different sizes. D) Ca^2+^ signal and PI intake in NIH-3T3 cells treated 3 hours with RSL3 in the presence or not of PEGs of different sizes. Each data point represents the mean of at least six replicas from three independent confocal microscopy experiments. At least 100 cells were analyzed for each replica. Concentrations: RSL3 (2 µM), PEGs (10 mM). PEG sizes: 400 (0.56 nm), 600 (0.69 nm), 1000 (0.94 nm), 2000 (1.6 nm), 4000 (1.8 nm), 6000 (2.3 nm) and 8000 (2.7 nm).

**Figure S3: Effect of PEGs on the time course of cell death in cells treated with different concentrations of RSL3.** A) NIH-3T3 cells, B) HT-1080 cells and C) Mda-157 cells. Each data point represents the mean of three independent Incucyte experiments. D) Cell death heat map of the effect of different PEGs on the extension of cell death in NIH-3T3 and HT-1080 cells (12 hours) and Mda-157 (24 hours) treated with different concentrations of RSL3.

**Figure S4: CHMP4B puncta formation in RSL3-treated cells to protect from cell death via membrane repair.** A-C) Representative images of HT-1080, Mda-157, and MEF cells transiently transfected with CHMP4B-eGFP and treated with RSL3, and monitored for CHMP4B puncta formation. Scale bar, 20 µm. D-F) Kinetics of the appearance of CHMP4B puncta in the corresponding cells, treated or not with RSL3. (n = 8 cells).

**Figure S5: Early increase of cytosolic Ca^2+^ upon Erastin-1 treatment does not correlate with CHMP4B puncta formation.** A) Time lapse confocal images of NIH-3T3 cells transiently transfected with CHMP4B-GFP and treated with Erastin-1, and monitored for CHMP4B puncta formation. Scale bar, 15 µm. Pictures are representative of at least three independent experiments. B) Kinetics of appearance of CHMP4B puncta and increase of Fluo-4 AM signal and cell rounding in Erastin-1 treated cells (n = 7 cells). Plots show the average temporal relationships between normalized mCherry fluorescence intensity standard deviation, cell rounding and Fluo-4 AM fluorescence intensity. Interval time between measurements was 5 minutes. C) The time delay between the appearance of CHMP4B puncta, Ca^2+^ signal and cell rounding. t_50_ of each event was calculated from individual curves obtained per single cells (shown in B). These values correspond to the time at 50% of the maximum signal and were plotted as lag time with respect to the appearance of CHMP4B puncta.

**Figure S6: Cytokine profile of ferroptotic cells.** A) H441 cells, B) MEF cells. In A and B, cytokines that are upregulated are highlighted in red and those that are downregulated in blue. C) Effect of CHMP4 depletion on the cytokine profile of ferroptotic cells. Summary of the cytokines that are regulated in A-C are shown in the left. In C, cytokines that are differently regulated upon CHMP4B depletion are highlighted in green. NIH-3T3 cells were treated with Erastin-1 (10 µM) for 24 hours. Supernatants were filtered and the cytokine composition was analyzed by the proteome profiler mouse XL cytokine Array kit.
