# Supplementary figures and images for: Ferroptotic pores induce Ca2+ fluxes and ESCRT-III activation to modulate cell death kinetics
Source: Cell Death Differ. 2020 Dec 17;28(5):1644–57. doi: 10.1038/s41418-020-00691-x (PMC8167089; doi:10.1038/s41418-020-00691-x)

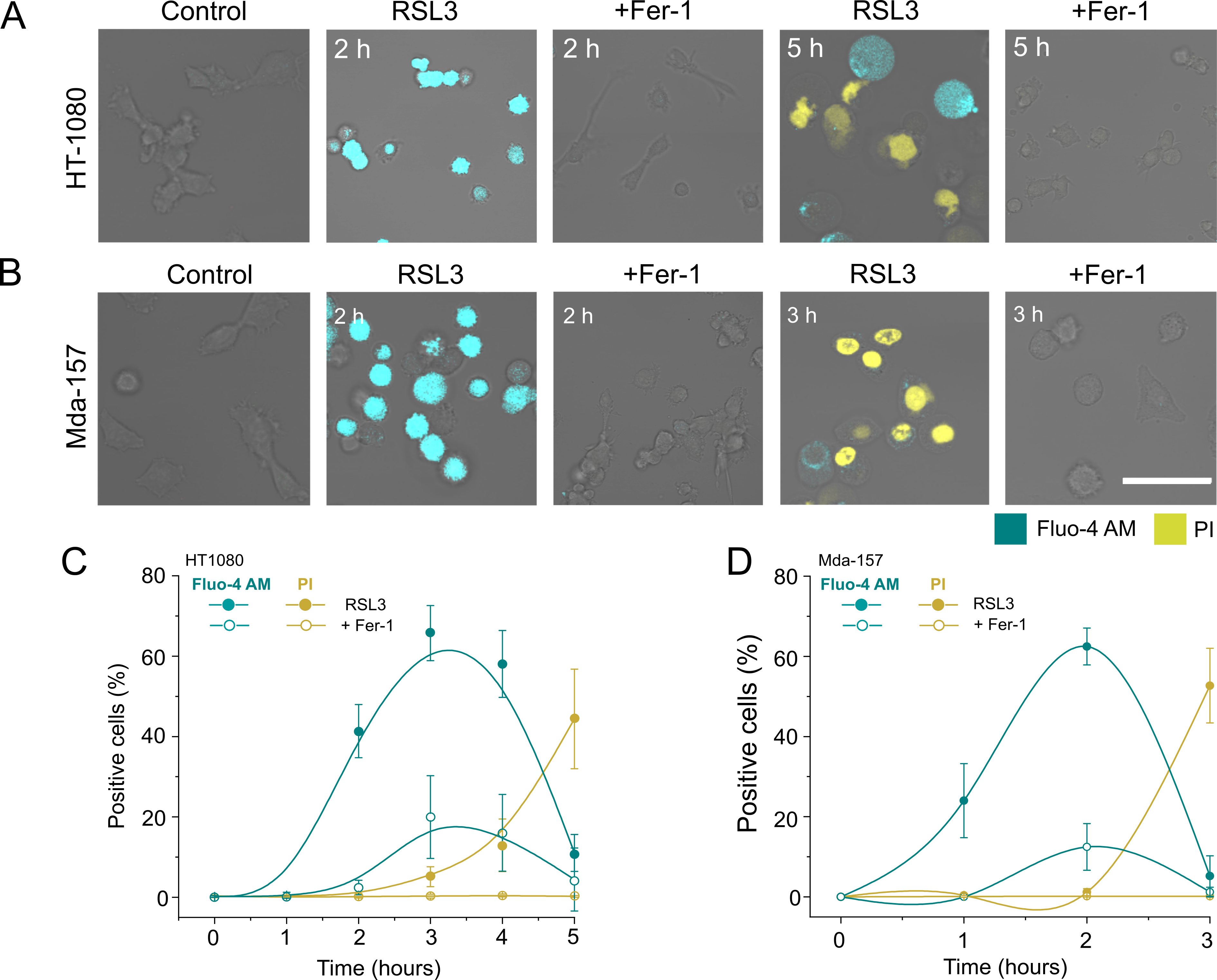

Supplement: Supplementary file 2 — Figure S1 [file 41418_2020_691_MOESM2_ESM.png]

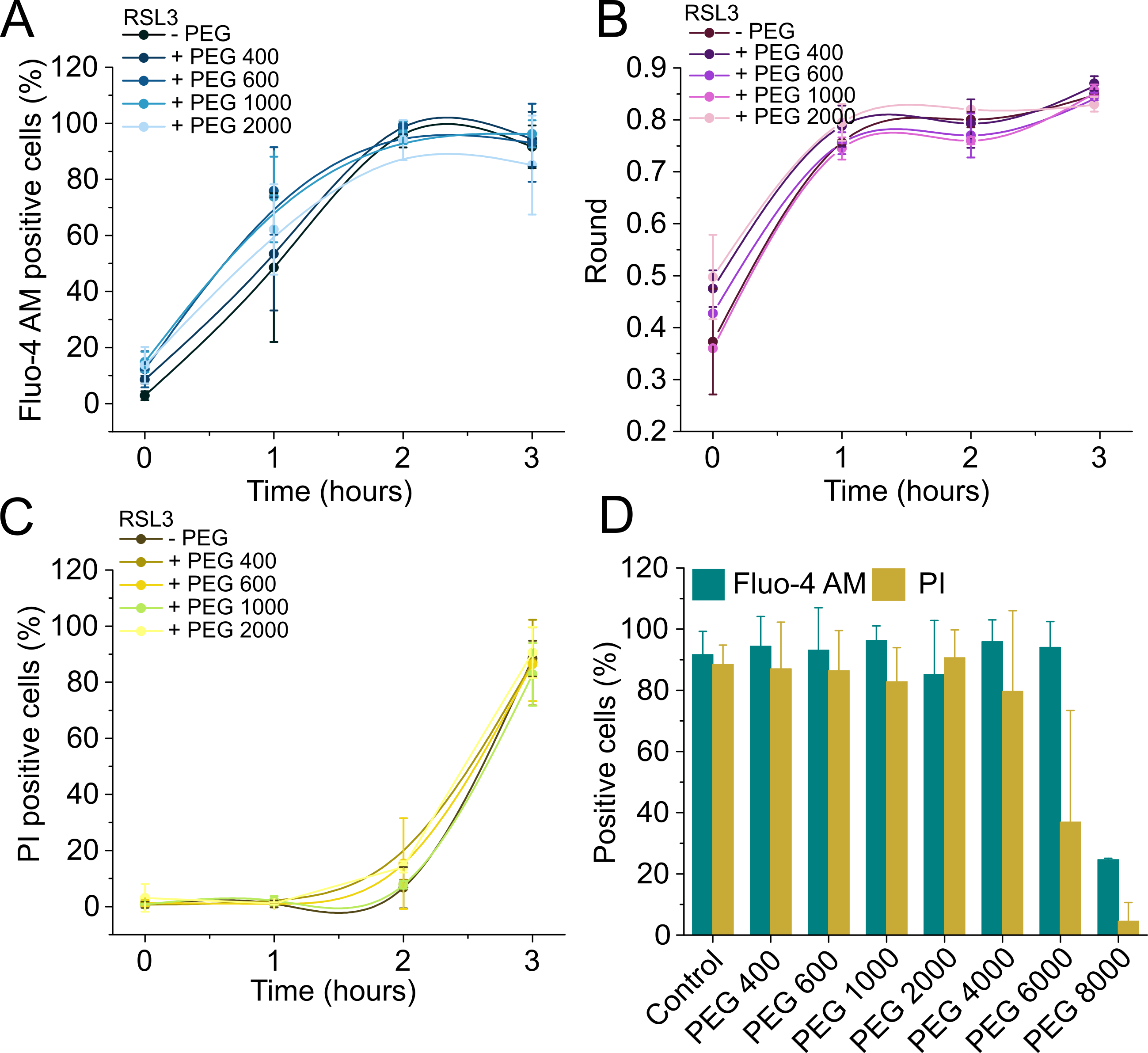

Supplement: Supplementary file 3 — Figure S2 [file 41418_2020_691_MOESM3_ESM.png]

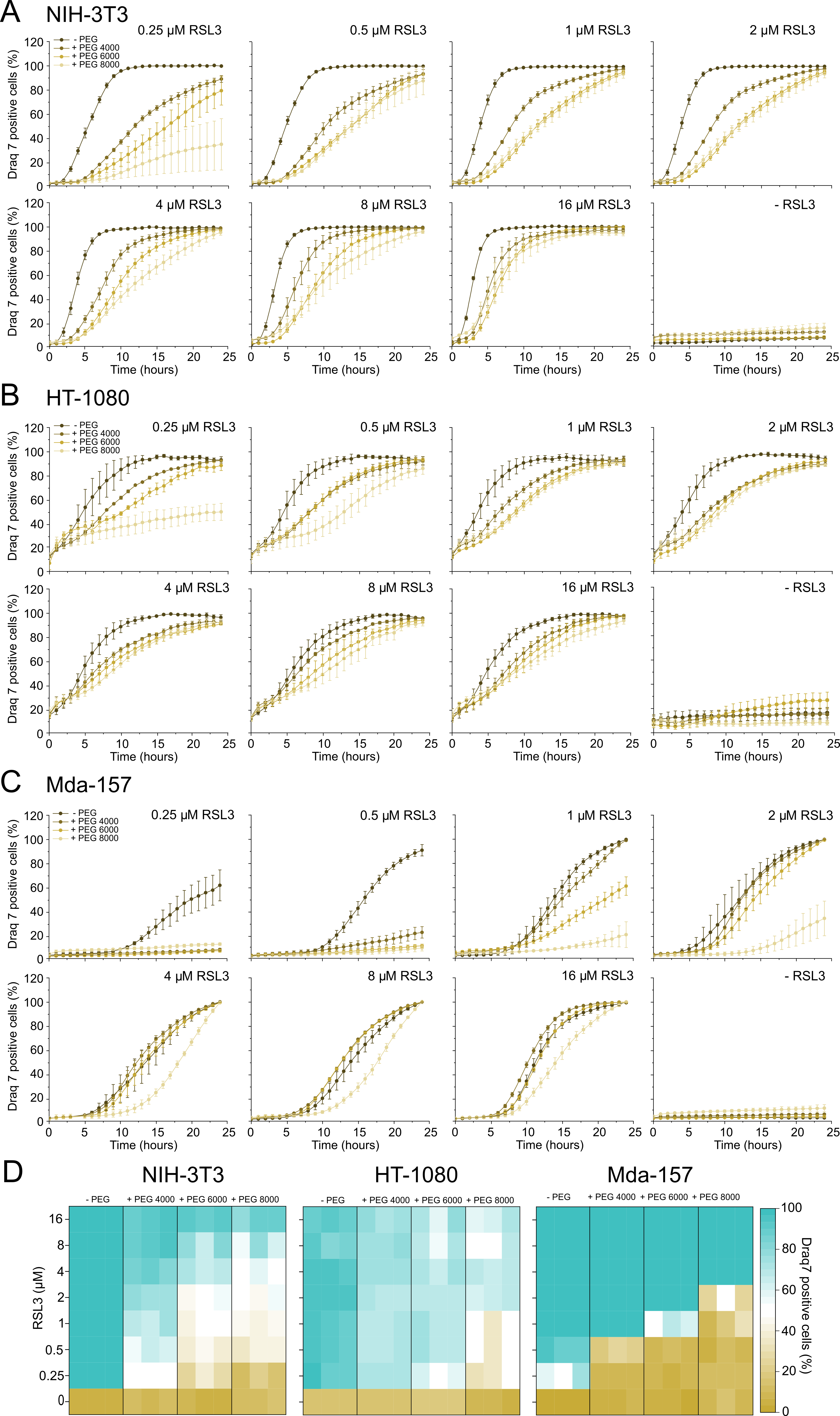

Supplement: Supplementary file 4 — Figure S3 [file 41418_2020_691_MOESM4_ESM.png]

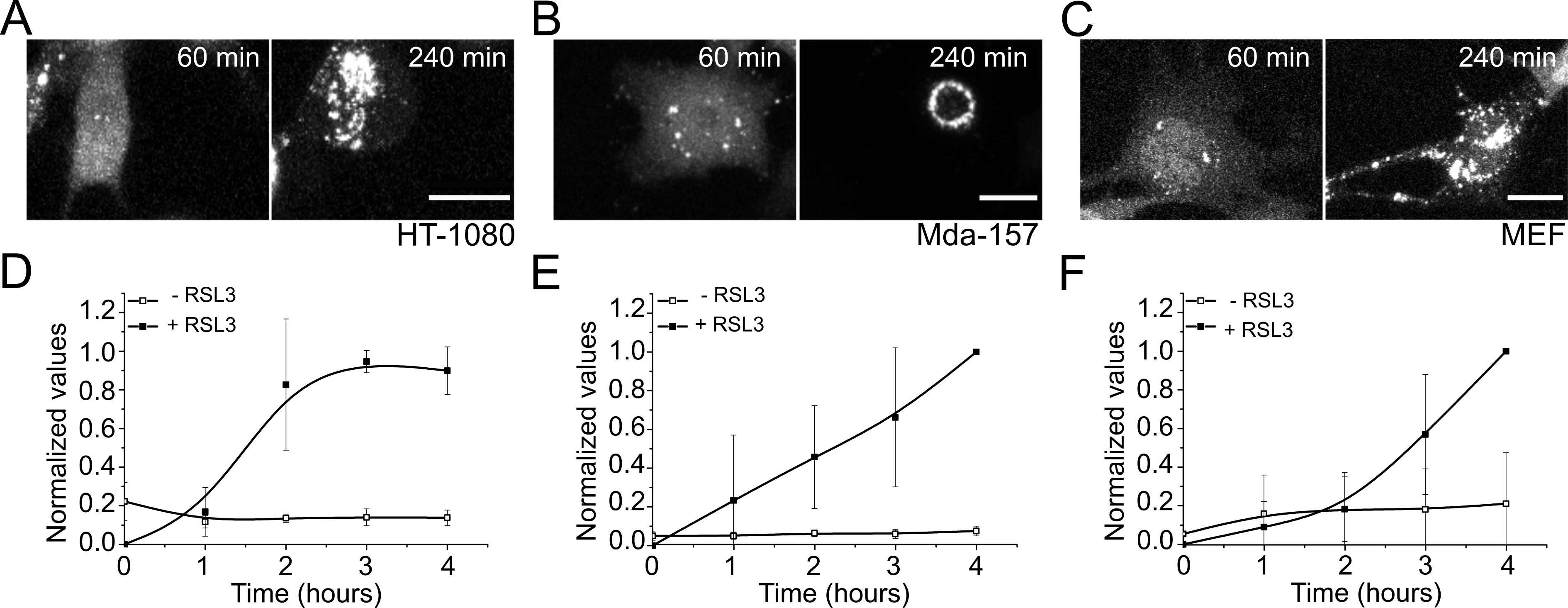

Supplement: Supplementary file 5 — Figure S4 [file 41418_2020_691_MOESM5_ESM.png]

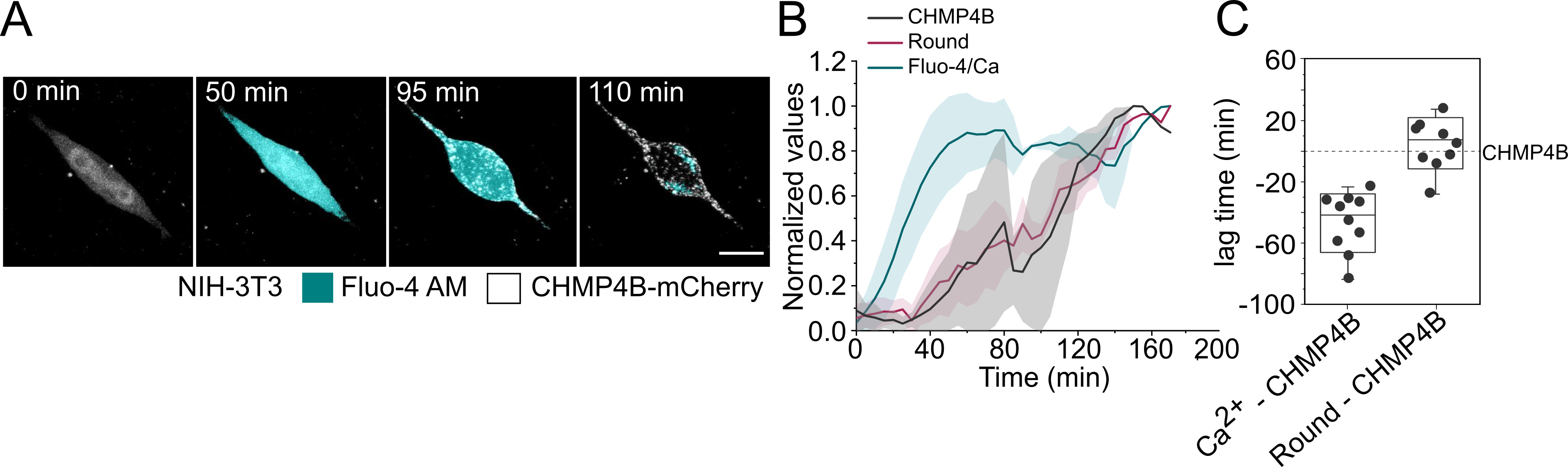

Supplement: Supplementary file 6 — Figure S5 [file 41418_2020_691_MOESM6_ESM.png]

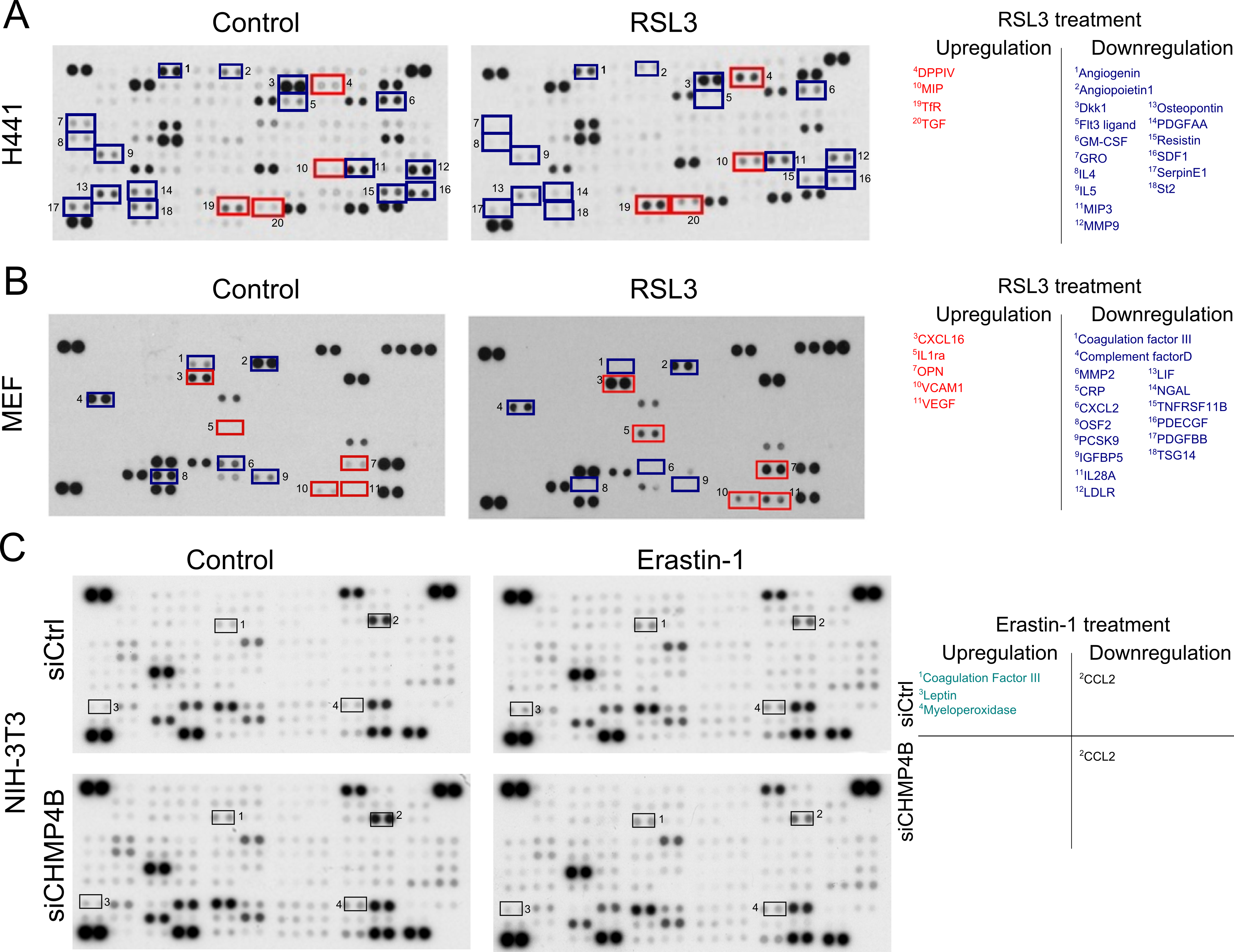

Supplement: Supplementary file 7 — Figure S6 [file 41418_2020_691_MOESM7_ESM.png]
